# Supplementary material for: Divergent metabolic programmes control two populations of MAIT cells that protect the lung
Source: Nat Cell Biol. 2023 May 25;25(6):877–91. doi: 10.1038/s41556-023-01152-6 (PMC10264248; doi:10.1038/s41556-023-01152-6)
Supplement: Supplementary file 1 — Supplementary Note 1. Synthesis of compounds. [file 41556_2023_1152_MOESM1_ESM.pdf]

nature cell biology

Article

https://doi.org/10.1038/s41556-023-01152-6

Divergent metabolic programmes control two populations of MAIT cells that protect the lung

In the format provided by the authors and unedited

Supplementary Note 1: Synthesis of compounds

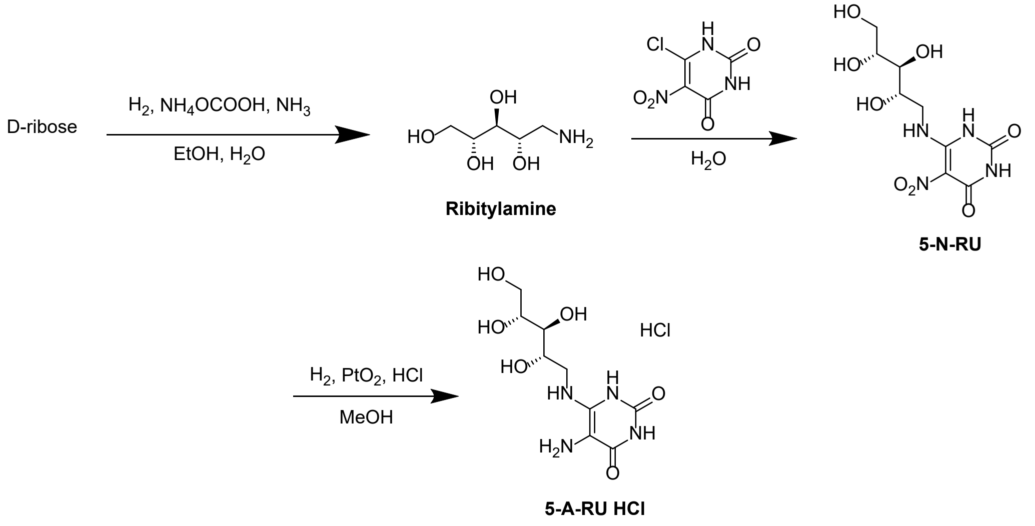

D-ribitylamine D-ribose (3.6 g, 24 mmol) was added to 90 mL of saturated aqueous ammonia and 200 mL of saturated ammonium formate in ethanol. 4 mL of Raney nickel slurry were added, then the reaction was shaken under 45 psi of hydrogen for 18 hr. The reaction was filtered over Celite to remove the catalyst, then concentrated under vacuum. The crude green syrup was redissolved in water, then applied to an Amberlite 120 column. The flow through was reapplied 2X, then the column was washed 2X with water and eluted 3X with saturated aqueous ammonia. The eluent was concentrated with ethanol under vacuum. The crude product was redissolved in water and acetonitrile, then lyophilized to give the product (3.4 g, 93% yield) as a beige, hygroscopic powder. The ribitylamine was dissolved in water and aliquoted at 91 mg/mL, then stored at -20° C. Adapted from Dangerfield et al(1).

5-N-RU. 6-chloro-5-nitropyrimidine-2,4(1H,3H)-dione (77 mg, 0.4 mmol) and D-ribitylamine (0.18 g, 1.2 mmol) in 2 mL of water were combined in a vial, which was sealed and heated to 190° C for 15 min with microwave irradiation. The reaction was diluted with 2 mL of water, filtered, then purified by RP-HPLC (Waters Atlantis T3 30x150 mm column, water/acetonitrile, 20 mL/min flow rate, 0.9 mL injection volume, 5 min 0% B, 10 min 0-50% B, 5 min 50% B). The fractions containing product were combined and lyophilized to give 63 mg of product (52% yield).

5-A-RU HCl (adapted from Li, *et al*. (2)). 5-N-RU (56 mg, 0.18 mmol) was dissolved in 10 mL of methanol. 0.2 mL of 12.1 N aqueous HCl and 5 mg of platinum (IV) oxide were added, then the reaction was shaken under 45 psi of hydrogen, excluding light as much as possible, for 18 hr. The reaction was filtered over Celite to remove catalyst, then concentrated under vacuum. The crude product was redissolved in water and acetonitrile, then lyophilized to give 56 mg of product as a red syrup (quant. yield).

1. Dangerfield EM, Plunkett CH, Win-Mason AL, Stocker BL, Timmer MS. Protecting-group-free synthesis of amines: synthesis of primary amines from aldehydes via reductive amination. J Org Chem. 2010;75(16):5470-7.
2. Li K, Vorkas CK, Chaudhry A, Bell DL, Willis RA, Rudensky A, et al. Synthesis, stabilization, and characterization of the MR1 ligand precursor 5-amino-6-D-ribitylaminouracil (5-A-RU). PLoS One. 2018;13(2):e0191837.
