# Supplementary material for: Divergent metabolic programmes control two populations of MAIT cells that protect the lung
Source: Nat Cell Biol. 2023 May 25;25(6):877–91. doi: 10.1038/s41556-023-01152-6 (PMC10264248; doi:10.1038/s41556-023-01152-6)
Supplement: Supplementary file 2 — Reporting Summary [file 41556_2023_1152_MOESM2_ESM.pdf]

nature portfolio

Corresponding author(s): Mitchell Kronenberg, Thomas Riffelmacher

Last updated by author(s): April 5th 2023

Reporting Summary

Nature Portfolio wishes to improve the reproducibility of the work that we publish. This form provides structure for consistency and transparency in reporting. For further information on Nature Portfolio policies, see our Editorial Policies and the Editorial Policy Checklist.

Statistics

For all statistical analyses, confirm that the following items are present in the figure legend, table legend, main text, or Methods section.

n/a	Confirmed	
☐	☒	The exact sample size (*n*) for each experimental group/condition, given as a discrete number and unit of measurement
☐	☒	A statement on whether measurements were taken from distinct samples or whether the same sample was measured repeatedly
☐	☒	The statistical test(s) used AND whether they are one- or two-sided<br>*Only common tests should be described solely by name; describe more complex techniques in the Methods section.*
☒	☐	A description of all covariates tested
☐	☒	A description of any assumptions or corrections, such as tests of normality and adjustment for multiple comparisons
☐	☒	A full description of the statistical parameters including central tendency (e.g. means) or other basic estimates (e.g. regression coefficient) AND variation (e.g. standard deviation) or associated estimates of uncertainty (e.g. confidence intervals)
☐	☒	For null hypothesis testing, the test statistic (e.g. F, t, r) with confidence intervals, effect sizes, degrees of freedom and P value noted<br>*Give P values as exact values whenever suitable.*
☒	☐	For Bayesian analysis, information on the choice of priors and Markov chain Monte Carlo settings
☒	☐	For hierarchical and complex designs, identification of the appropriate level for tests and full reporting of outcomes
☒	☐	Estimates of effect sizes (e.g. Cohen's *d*, Pearson's *r*), indicating how they were calculated

Our web collection on statistics for biologists contains articles on many of the points above.

Software and code

Policy information about availability of computer code

Data collection	FACS DIVA 8.0 (BD Biosciences) was used for all Flow Cytometry data acquisition. Zeiss Zen Blue v3 was used for confocal image data aquisition. BCL (BinaryBaseCall) were used as input to create FASTQ files with cellranger mkfastq v 6.1.2
Data analysis	FlowJo v 10.8.1 (TreeStar Inc) was used for flow cytometry data analysis. Excel v16 and Graphpad Prism v9.0 were used for statistical tests, data analysis and graph plotting. Automated image quantification: Imaris 10 (Bitplane), using the spot detection algorithm. scRNA-seq data: FastQ file alignment: refdata-gex-mm10-2020-A with cellranger count v 6.1.2. Analysis via Seurat v 4.1.1 and Seurat's FindAllMarkers function and Seurat's AddModuleScore function. For Mass spectometry analysis, mass isotopologue distributions and fractional contributions of metabolites were quantified using TraceFinder 3.3.

For manuscripts utilizing custom algorithms or software that are central to the research but not yet described in published literature, software must be made available to editors and reviewers. We strongly encourage code deposition in a community repository (e.g. GitHub). See the Nature Portfolio guidelines for submitting code & software for further information.

Data

Policy information about availability of data

All manuscripts must include a data availability statement. This statement should provide the following information, where applicable:

- Accession codes, unique identifiers, or web links for publicly available datasets
- A description of any restrictions on data availability
- For clinical datasets or third party data, please ensure that the statement adheres to our policy

Bulk and sc-RNA-seq data that support the findings of this study have been deposited in the Gene Expression Omnibus (GEO) under accession code GSE226524. Publicly available RNA-seq data was used from the C7 immunological database: Accession codes GSE1000002_1582_200_UP, GSE1000002_1582_200_DN, 20220131-155727-scavenger_receptors

Human research participants

Policy information about studies involving human research participants and Sex and Gender in Research.

Reporting on sex and gender	N/A
Population characteristics	N/A
Recruitment	N/A
Ethics oversight	N/A

Note that full information on the approval of the study protocol must also be provided in the manuscript.

Field-specific reporting

Please select the one below that is the best fit for your research. If you are not sure, read the appropriate sections before making your selection.

☒ Life sciences ☐ Behavioural & social sciences ☐ Ecological, evolutionary & environmental sciences

For a reference copy of the document with all sections, see nature.com/documents/nr-reporting-summary-flat.pdf

Life sciences study design

All studies must disclose on these points even when the disclosure is negative.

Sample size	No sample size calculations were performed. Sample size was determined based on the magnitude and variation of measurable differences between groups, as well as the feasibility of performing highly technical experiments with rare cell populations. Sample sizes are indicated in figure legends. All data points represent individual mice and mice from genetically distinct comparison groups (e.g. cre+ vs cre-) are littermates from the same breeders unless indicated otherwise. This limits sample size, in addition to ethical considerations when determining necessary power of animal experiments.
Data exclusions	Post-sequencing, stringent, pre-established quality controls were applied: For mouse single cell RNA sequencing analysis, cells with less than 200 genes and more than 2,500 genes were discarded. Cells with more than 5% UMIs coming from mitochondrial genes were filtered out. Genes expressed in less than 3 cells were ignored. No other data was excluded in any studies.
Replication	All experiments reported here with the exception of scRNAseq experiment (n=1) have been repeated independently 2 or more times, and exact number of individual experiments are indicated in legends. In many instances data from individual experiments have been pooled in the representations, and this is indicated in legends. All experiments were reproducible.
Randomization	Randomization was not applied. Mice were gender and aged matched and breedings were setup so that every experimental group is represented in each litter.
Blinding	The investigators were not blinded during data collection. In most cases, data was quantified in an unsupervised manner/by data analysis software. In many cases (scRNA-seq, imaging) samples were processed and analyzed by separate scientists via automated algorythms and bioinformatic analysis was done by separate bioinformaticians.

Reporting for specific materials, systems and methods

We require information from authors about some types of materials, experimental systems and methods used in many studies. Here, indicate whether each material, system or method listed is relevant to your study. If you are not sure if a list item applies to your research, read the appropriate section before selecting a response.

Materials & experimental systems

n/a	Involved in the study
☐	☒ Antibodies
☒	☐ Eukaryotic cell lines
☒	☐ Palaeontology and archaeology
☐	☒ Animals and other organisms
☒	☐ Clinical data
☒	☐ Dual use research of concern

Methods

n/a	Involved in the study
☒	☐ ChIP-seq
☐	☒ Flow cytometry
☒	☐ MRI-based neuroimaging

Antibodies

Antibodies used

MR1 tetramers loaded with either 5-OP-RU or 6-FP were obtained from the NIH Tetramer Facility. Antibodies with clone, dilution, catalogue number and supplier indicated in parentheses: anti-mouse CD16/32 (2.4G2, Cat#553142, 1:500, BD Bioscience), anti-mouse Sdc1 (281-2, 1:200, Cat# 564068 BDBioscience), anti-mouse CD36 (HM36, 1:200, Cat#102608, Biolegend), anti-mouse CD45 (30-F11, 1:800, Cat#564225 BDBioscience); anti-mouse IgD (clone 11-26c.2a, 1:200, Cat#564273 BDBioscience); anti-mouse Klrg1 (clone 2F1/KLRG1, 1:200, 25-5893-82 Invitrogen); anti-mouse gdTCR (clone GL3, 1:200, Cat#553178 BDBioscience); anti-mouse TER-119 (TER-119, 1:200, Cat#116204 Biolegend) anti-mouse CD127 (clone SB/199, 1:200, Cat#562419 BD Bioscience); anti-mouse CD8a (clone 53-6.7, 1:400, Cat#47-0081-82 eBioscience); anti-mouse CD8b (H35-17.2, 1:400, Cat#741992 BDBioscience), anti-mouse EpCAM (clone G8.8, 1:200, Cat#13-5791 eBioscience); anti-mouse IFN-g(clone XMG1.2, 1:400, Cat# 505831 Biolegend); anti-mouse TNF (clone MP6-XT22, 1:400); anti-mouse IL-17A (clone TC11-18H10, 1:400, Cat# 506914 Biolegend); anti-mouse CD69 (clone H1.2F3, 1:200, Cat# 104530 Biolegend); anti-mouse CD44 (IM7, 1:200, Cat# 586116 BDBioscience), anti-mouse Ly6A/E (D7, 1:200, Cat# 108110 Biolegend), anti-mouse Icos (C398.4a, 1:200, Cat# 313520 Biolegend), anti-mouse T-bet (clone O4-46, 1:100 Cat# 562467 BDBioscience); anti-mouse RORgT (clone B2D, 1:100, Cat# 46-6981-82 eBioscience); anti-mouse Ly6G (clone 1A8, 1:400, Cat# 127639 Biolegend); anti-mouse CD11b (M1/70, 1:400, Cat# 561114 BDBioscience); anti-mouse CD45R/B220 (RA3-6B2, 1:200 Cat# 552771 BDBioscience); anti-mouse CD11c (N418, 1:200, Cat# 48-0114-80 eBioscience); anti-mouse TCRb(H57-597, 1:200, Cat# 47-5961-82 eBioscience), anti-mouse Ki67 (B56, 1:200, Cat# 561281 BDBioscience); anti-mouse CD80 (16-10A1, 1:200, Cat# 553768 BDBioscience); anti-mouse CD86 (GL1, 1:200); anti-mouse MR1 (26.5, 1:200, Cat# 361107 Biolegend); anti-mouse Granzyme B (GB11, 1:400, Cat# GRB05 Invitrogen); anti-mouse CD103 (2e7, 1:200, Cat# 749393, BDBioscience); anti-mouse siglec F (E50-2440 1:200, Cat# 565527 BDBioscience), anti-mouse Tom20 antibody (D8T4N, 1:400, Cat#13929, Cell Signaling).

Validation

1. anti-mouse CD16/32 antibody: The manufacturer states that specificity has been validated for Flow Cytometry: https://www.bdbiosciences.com/en-us/products/reagents/flow-cytometry-reagents/research-reagents/single-color-antibodies-ruo/purified-rat-anti-mouse-cd16-cd32-mouse-bd-fc-block.553142
2. anti-mouse Sdc1 antibody: The manufacturer states that specificity has been validated for Flow Cytometry:https://www.bdbiosciences.com/en-us/products/reagents/flow-cytometry-reagents/research-reagents/single-color-antibodies-ruo/bv650-rat-anti-mouse-cd138.564068
3. anti-mouse CD36 antibody: https://www.biolegend.com/en-us/products/alexa-fluor-488-anti-mouse-cd36-antibody-3311?GroupID=BLG10704
4. anti-mouse CD45 antibody: https://www.bdbiosciences.com/en-us/products/reagents/flow-cytometry-reagents/research-reagents/single-color-antibodies-ruo/bv786-rat-anti-mouse-cd45.564225
5. anti-mouse IgD antibody: https://www.bdbiosciences.com/en-sg/products/reagents/flow-cytometry-reagents/research-reagents/single-color-antibodies-ruo/percp-cy-5-5-rat-anti-mouse-igd.564273
6. anti-mouse Klrg1 antibody: https://www.thermofisher.com/antibody/product/KLRG1-Antibody-clone-2F1-Monoclonal/25-5893-82
7. anti-mouse gdTCR antibody: https://www.bdbiosciences.com/en-us/products/reagents/flow-cytometry-reagents/research-reagents/single-color-antibodies-ruo/pe-hamster-anti-mouse-t-cell-receptor.561997
8. anti-mouse TER-119 antibody: https://www.biolegend.com/en-us/products/biotin-anti-mouse-ter-119-erythroid-cells-antibody-1864
9. anti-mouse CD127 antibody: https://www.bdbiosciences.com/en-ca/products/reagents/flow-cytometry-reagents/research-reagents/single-color-antibodies-ruo/pe-cf594-rat-anti-mouse-cd127.562419
10.anti-mouse CD8a antibody: https://www.thermofisher.com/antibody/product/CD8a-Antibody-clone-53-6-7-Monoclonal/47-0081-82
11.anti-mouse CD8b antibody: https://www.bdbiosciences.com/en-at/products/reagents/flow-cytometry-reagents/research-reagents/single-color-antibodies-ruo/BUV805-Rat-Anti-Mouse-CD8b.741992
12. anti-mouse EpCAM antibody: https://www.thermofisher.com/antibody/product/CD326-EpCAM-Antibody-clone-G8-8-Monoclonal/13-5791-82
13. anti-mouse Ifng antibody: The manufacturer states that specificity has been validated for Flow Cytometry:https://www.biolegend.com/fr-fr/products/brilliant-violet-650-anti-mouse-ifn-gamma-antibody-7681
14. anti-mouse Tnf antibody
15. anti-mouse IL-17A antibody: The manufacturer states that specificity has been validated for Flow Cytometry:https://www.biolegend.com/en-us/products/alexa-fluor-700-anti-mouse-il-17a-antibody-3539?GroupID=GROUP24
16. anti-mouse Cd69 antibody: The manufacturer states that specificity has been validated for Flow Cytometry:https://www.biolegend.com/en-us/products/brilliant-violet-605-anti-mouse-cd69-antibody-7864?GroupID=BLG10515
17. anti-mouse Cd44 antibody: The manufacturer states that specificity has been validated for Flow Cytometry:https://www.bdbiosciences.com/en-us/products/reagents/flow-cytometry-reagents/research-reagents/single-color-antibodies-ruo/pe-rat-anti-mouse-cd44.561860
18. anti-mouse Ly6A/E antibody: The manufacturer states that specificity has been validated for Flow Cytometry:https://www.biolegend.com/en-us/products/pe-cyanine5-anti-mouse-ly-6a-e-sca-1-antibody-229?GroupID=BLG2524

19. anti-mouse Icos antibody: The manufacturer states that specificity has been validated for Flow Cytometry:https://www.biolegend.com/fr-fr/products/pe-cyanine7-anti-human-mouse-rat-cd278-icos-antibody-6908
20. anti-mouse T-bet antibody: The manufacturer states that specificity has been validated for Flow Cytometry:https://www.bdbiosciences.com/en-us/products/reagents/flow-cytometry-reagents/research-reagents/single-color-antibodies-ruo/pe-cf594-mouse-anti-t-bet.562467
21. anti-mouse Rorgt antibody: The manufacturer states that specificity has been validated for Flow Cytometry:https://www.thermofisher.com/antibody/product/ROR-gamma-t-Antibody-clone-B2D-Monoclonal/46-6981-82
22. anti-mouse Ly6G antibody: The manufacturer states that specificity has been validated for Flow Cytometry: https://www.biolegend.com/en-us/products/brilliant-violet-605-anti-mouse-ly-6g-antibody-12244?GroupID=BLG7234
23. anti-mouse Cd11b antibody: The manufacturer states that specificity has been validated for Flow Cytometry:https://www.bdbiosciences.com/en-us/products/reagents/flow-cytometry-reagents/research-reagents/single-color-antibodies-ruo/percp-cy-5-5-rat-anti-cd11b.561114
24. anti-mouse Cd45/B220 antibody: The manufacturer states that specificity has been validated for Flow Cytometry:https://www.bdbiosciences.com/en-us/products/reagents/flow-cytometry-reagents/research-reagents/single-color-antibodies-ruo/percp-cy-5-5-rat-anti-mouse-cd45r-b220.552771
25. anti-mouse Cd11c antibody: The manufacturer states that specificity has been validated for Flow Cytometry:https://www.thermofisher.com/antibody/product/42-0114-82.html?ef_id=CjwKCAjw5pShBhB_EiwAvmnNV0miRHViUP_dgGczGVPovK4rtZDNtuJVskFrnG1WY9useSwt-INpYRoCs80QAvD_BwE:G:s&s_kwcid=AL!3652!3!459737518508!!!g!!!10950825775!106531320406&cid=bid_pca_aup_r01_co_cp1359_pjt0000_bid00000_0se_gaw_dy_pur_con&gclid=CjwKCAjw5pShBhB_EiwAvmnNV0miRHViUP_dgGczGVPovK4rtZDNtuJVskFrnG1WY9useSwt-INpYRoCs80QAvD_BwE
26. anti-mouse TCRb antibody: The manufacturer states that specificity has been validated for Flow Cytometry:https://www.thermofisher.com/antibody/product/TCR-beta-Antibody-clone-H57-597-Monoclonal/47-5961-82
27. anti-mouse Ki67 antibody: The manufacturer states that specificity has been validated for Flow Cytometry:https://www.bdbiosciences.com/en-us/products/reagents/flow-cytometry-reagents/research-reagents/single-color-antibodies-ruo/v450-mouse-anti-ki-67.561281
28. anti-mouse Cd80 antibody: The manufacturer states that specificity has been validated for Flow Cytometry:https://www.bdbiosciences.com/en-us/products/reagents/flow-cytometry-reagents/research-reagents/single-color-antibodies-ruo/fitc-hamster-anti-mouse-cd80.553768
29. anti-mouse Cd86 antibody: The manufacturer states that specificity has been validated for Flow Cytometry:https://www.biolegend.com/en-us/products/pe-anti-mouse-cd86-antibody-256?GroupID=BLG10719
30. anti-mouse Mr1 antibody: The manufacturer states that specificity has been validated for Flow Cytometry:https://www.biolegend.com/fr-ch/products/apc-anti-human-mouse-rat-mr1-antibody-9373
31. anti-mouse GzmB antibody: The manufacturer states that specificity has been validated for Flow Cytometry:https://www.thermofisher.com/antibody/product/Granzyme-B-Antibody-clone-GB11-Monoclonal/GRB05
32. anti-mouse Cd103 antibody: The manufacturer states that specificity has been validated for Flow Cytometry:https://www.bdbiosciences.com/en-nz/products/reagents/flow-cytometry-reagents/research-reagents/single-color-antibodies-ruo/buv737-hamster-anti-mouse-cd103.749393
32. anti-mouse SiglecF antibody: The manufacturer states that specificity has been validated for Flow Cytometry:https://www.bdbiosciences.com/en-us/products/reagents/flow-cytometry-reagents/research-reagents/single-color-antibodies-ruo/apc-cy-7-rat-anti-mouse-siglec-f.565527
33. anti-mouse Tom20 antibody: The manufacturer states that specificity has been validated for Immunofluorescence:https://www.cellsignal.com/products/primary-antibodies/tom20-antibody/13929?Ntk=Products&Ntt=13929&gclid=CjwKCAjw5pShBhB_EiwAvmnNV9SFjaRKitrlHkhZLY0MERMoDN-6nc_MfziMG9MGlYpQ5CqqQK8oAhoCuz0QAvD_BwE&gclsrc=aw.ds

Animals and other research organisms

Policy information about studies involving animals; ARRIVE guidelines recommended for reporting animal research, and Sex and Gender in Research

Laboratory animals	C57BL/6J mice, Atg5f/f, Vhlf/f, Hif1af/f and dLck-Cre mice were purchased from Jackson Laboratory and crossed to generate Atg5f/f dLck-Cre, Vhlf/f dLck-Cre and Vhlf/f Hif1af/f dLck-Cre mice. Heterozygous Cre mice were used, together with littermate controls, in all experiments. All mice were on the C57BL/6J background and used at 6-12 weeks of age. B6;CBA-Tg(Tbx21-cre)1Dlc/J (Tbet-cre) were bred with B6.Cg-Gt(ROSA)26Sortm14(CAG-tdTomato)Hze/J (Td-tomato) mice (from Jackson Laboratories) to generate the T-bet fate mapping line. B6.129(SJL)-Il17atm1.1(icre)Stck/J (IL-17A-YFP fate-map) mice were purchased from Jackson Laboratory. ¬ Ja18-/-(Traj18-/-) mice were generated as described previously (see methods section). Mr1-/- mice were kindly provided by Dr. Gilfillan (Washington University, St. Louis)(see methods for detail). C57BL/6-TgCAG-RFP/EGFP/Map1lc3b transgenic mice were from Jackson Laboratory. Mice were group-housed under a standard rodent chow (Pico Lab Diet 20, #5053) at ambient temperature (68°F), 50% humidity in average and a 12 hour light-dark cycle in individually ventilated cages. Food and water were provided ad libitum.
Wild animals	This study did not include wild animals
Reporting on sex	Both male and female mice were used in these studies. No obvious sex based difference was evident in these experiments, but the data have not been specifically analyzed after disaggregation for sex.
Field-collected samples	No field collected samples were used in this study
Ethics oversight	All procedures were approved by the La Jolla Institute for Immunology or University of California San Diego Animal Care and Use Committee and are compliant with the ARRIVE standards.

Note that full information on the approval of the study protocol must also be provided in the manuscript.

Flow Cytometry

Plots

Confirm that:

☒ The axis labels state the marker and fluorochrome used (e.g. CD4-FITC).

☒ The axis scales are clearly visible. Include numbers along axes only for bottom left plot of group (a 'group' is an analysis of identical markers).

☒ All plots are contour plots with outliers or pseudocolor plots.

☒ A numerical value for number of cells or percentage (with statistics) is provided.

Methodology

Sample preparation	Lung tissue was digested with spleen dissociation medium (Stemcell), and mechanically dissociated using a GentleMacs Dissociator (Miltenyi). Cells were then strained though a 70 µm filter and washed with HBSS supplemented with 10% fetal bovine serum (FBS) followed by RBC lysis. For adoptive transfer experiments, Penicillin-Streptomycin (Gibco) was added to media throughout the experiment. For staining of cell surface molecules, cells were suspended in staining buffer (PBS, 1% bovine serum albumin (BSA), and 0.01% NaN3) and stained using PE- or APC-conjugated MR1 tetramers at a dilution of 1:300 in staining buffer for 30 minutes at room temperature followed by staining with fluorochrome-conjugated antibody at 0.1–1 µg/107 cells. Cells were stained with Live/Dead Yellow (ThermoFisher) at 1:500 and blocked with 2.4G2 antibody at 1:500 and Free Streptavidin at 1:1000 for 15 min at 4℃, continued with surface antibody staining for 30 min at 4℃. For cytokine staining, cells were previously stimulated with 50 ng/ml of PMA and 1[illegible]g/ml of Ionomycin for 2h at 37℃ and then incubated in GolgiStop and GolgiPlug (both from BD Pharmingen) for 2 h at 37℃. For in vitro re-activation experiments, cultures were carried out for 18 hours without stimulation or with 5-OP-RU (1µM) and/or IL-2+IL-12+IL-18 (10ng/ml each). GolgiStop and GolgiPlug (both from BD PharMingen) were added for the last 2 hours. Following S. pneumoniae infection, cells were incubated in GolgiStop and GolgiPlug for 2 h at 37℃ with no restimulation. For intracellular staining, cells were fixed with CytoFix (BD) for 20 min, and permeabilized with Perm 1X solution (Thermo Fisher) overnight with antibodies for intracellular marker detection.
Instrument	Data were acquired on Fortessa and LSR-II cytometers and analyzed with FlowJo v10.8 (BD). Sorting was carried out on FACSAria(TM) Fusion and FACS Aria-3(TM) Cytometers (BD Biosciences)
Software	FACS DIVA 8.0 (BD Biosciences) was used for all Flow Cytometry data aquisition. Flowjo v10.8.1 was used for data analysis
Cell population abundance	Sorting efficiency was routinely controlled during sorting and didn't drop below 90%.
Gating strategy	Gating strategies are displayed in Extended Data Figure 1 +2

☒ Tick this box to confirm that a figure exemplifying the gating strategy is provided in the Supplementary Information.
